# Integrin Alpha V in Urine: A Novel Noninvasive Marker for Prostate Cancer Detection

**DOI:** 10.3389/fonc.2020.610647

**Published:** 2021-03-10

**Authors:** Marina Y. Zemskova, Maria V. Marinets, Andrey V. Sivkov, Julia V. Pavlova, Andrey N. Shibaev, Konstantin S. Sorokin

**Affiliations:** ^1^ G.K. Skryabin Institute of Biochemistry and Physiology of Microorganisms, Federal Research Center, Pushchino Center for Biological Research of the Russian Academy of Sciences, Pushchino, Russia; ^2^ Department of the Research, Prostagnost LLC, Moscow, Russia; ^3^ N.A. Lopatkin Research Institute of Urology and Interventional Radiology, Branch of FSBI National Medical Research Radiological Center, Ministry of Health of the Russian Federation, Moscow, Russia; ^4^ Department of Urology, M.F. Vladimirsky Moscow Regional Research and Clinical Institute (MONIKI), Moscow, Russia

**Keywords:** prostate cancer, urine, biomarker, integrin alpha V, cancer diagnostic marker, non-invasive cancer screening

## Abstract

Prostate cancer (PCa) diagnosis based on patient urine analysis provides non-invasive and promising method as compared to biopsy and a prostate-specific antigen (PSA) test. This study was conceived to investigate whether Integrin alpha V (ITGAV) protein is present in urine and assess the urinary ITGAV diagnostic potential for PCa. Materials and Methods: Urinary ITGAV expression was determined by Western blot analysis and quantified by ELISA in urine from men with PCa (n = 47), benign prostate hyperplasia (n = 42) and age-matched controls (n = 22). Results: The level of ITGAV protein was significantly lower in PCa urine samples as compared to those in the control group (p < 0.00001). The decrease of ITGAV in urine was highly predictive of PCa with 91.5% sensitivity, 91.4% specificity, 0.93 area under the ROC curve, and its specificity was better than that of serum PSA. Conclusion: Urinary ITGAV provides a novel noninvasive biomarker with high specificity.

## Introduction

Prostate cancer (PCa) is the primary cause of death in men all over the world, which is associated, in part, with the absence of relevant biomarkers for early diagnosis and treatment. Currently, the prostate specific antigen (PSA) is well-known and widely used biomarker for PCa diagnosis. However, this test based on elevated level of PSA in blood serum of PCa patients has a number of limitations related to low specificity and potential over-diagnosis. The false positive results (PSA > 4 ng/ml) have been observed in patients with benign prostatic hyperplasia (BPH) and prostatitis, while the low serum PSA values (less than 4 ng/ml) have been detected in patients having aggressive cancer with the Gleason score > 7 (1, 2). These limitations in accuracy in cancer prediction and monitoring require the urgent need of additional biomarkers, which could become a complement of serum PSA.

Unlike blood tests, urine analysis is a non-invasive approach to screen the prostate cancer. The urethra runs through the prostate gland and prostate fluid is mixed with urine, which allows for the estimation of prostate status. This, in turn, provides a potential for early PCa diagnosis in case if appropriate biomarkers are found. Genetic and epigenetic PCa-specific biomarkers in urine such as non-coding RNA of prostate cancer antigen-3 (PCA3), gene methylation of glutathione S-transferase P (GSTP1), and fusion transcripts for transmembrane protease serine-2 and ERG (TMPRSS2:ERG) have been developed and evaluated for the past decades (3). The single urinary PCA3 test has been approved so far for clinical use. Studies of microRNAs (miRNAs) and exosomes isolated from urine is another area to search for biomarkers, which could be promising in pre-biopsy prediction of PCa (3–6). Recently, integrated analysis of metabolomic and transcriptomic data obtained from urine samples of PCa patients, BPH, and healthy individuals revealed abnormal glutamate metabolism and tricarboxylic acid cycle in the prostate cancer (7). These pathway components can represent potential urine PCa biomarkers; nevertheless, the validation and adaptation of such a complex test to apply it in routine clinical practice are required.

Another rational approach to search for PCa specific biomarkers is based on prostate tissue immunohistochemical (IHC) analysis. The elevated level of the Engrailed-2 (EN2) protein biomarker was first discovered by IHC in PCa biopsies and then validated with the use of Western blot analysis and enzyme-linked immunosorbent assay (ELISA) of urine samples (8). The Homeobox protein EN2 is a transcription factor, which is predominantly expressed in malignant prostate tissues and detectable in urine samples of PCa patients with sensitivity and specificity of 66% and 88%, respectively. Urinary EN2 levels correlate with tumor volume and stage (9). Another known urinary protein biomarker, annexin A3 (ANXA3), belongs to a family of calcium and phospholipid binding proteins (10). The prognostic relevance of ANXA3 for PCa was validated by IHC in prostate tissues and the decreased level of ANXA3 was also found by western blot analysis in urine samples of prostate cancer patients (11, 12). Currently, the both urinary proteins, which are studied by many research groups, are promising candidates for their application as noninvasive PCa diagnostics.

Integrins are heterodimeric integral membrane proteins composed of alpha (α) and beta (β) subunits which are involved in cell surface adhesion and signaling pathways. These proteins were implicated in pathology of tumor progression and metastasis because of their involvement in multiple physiological functions of cell survival, migration, and invasion (13, 14). The integrin alpha-V (αv, ITGAV) preproprotein is proteolytically processed to produce the light and heavy chains composing the αv subunit. This subunit is associated with the beta 1, beta 3, beta 5, beta 6, and beta 8 subunits. A tissue microarray (TMA) of 1284 patient samples was used in a study with antibodies specific to the αvβ3, αvβ5, αvβ6, αvβ8, β3, and αv-pan to show that only the αvβ5 and αv-pan proteins were ubiquitously expressed in prostate cancer cells. Moreover, their expression varied depending on histopathologic localization and a cancer stage (15). There is still little knowledge about αv biological significance in prostate carcinoma since the majority of the previous studies were focused only on the αvβ3 and αvβ6 heterodimers (16, 17).

In this study, the αv (ITGAV) protein was quantified in urine samples from a standard clinical population with the use of western blot and ELISA in order to determine a urine ITGAV level and find out whether its specificity and sensitivity is significant for PCa detection. The results of this study were compared with those of serum PSA-based diagnostics.

## Materials and Methods

### Patient and Controls

The study includes patients who were observed in two medical institutions in Moscow. The research Protocol was approved by the institutes’ ethics committees. Before the study, participants signed an informed consent form. The study includes 42 patients with LUTS/BPH and 47 with prostate cancer, as well as 22 healthy volunteers (**Table S2**). We did not include patients with PCa who had undergone surgery, radiation treatment, or chemotherapy prior to the study.

All participants in the study had their urine sample taken (without prior prostate massage) before undergoing a prostate biopsy or surgery. Totally, 133 urine samples were collected. The urine samples were centrifuged at 3,000 rpm for 10 min to remove cells and debris. Later samples were divided into 1.5-ml Eppendorf tubes and kept at –80°C until their analysis. The urine samples were blinded to laboratory staff at the time of ITGAV measurement.

The first and the second passes of urine samples from healthy individuals of control group (53 to 67 years old) were collected into separate collection cups, centrifuged at 3,000 rpm for 10 min, and their supernatants were used for comparative analysis of protein levels by ELISA.

Some patients already had a known diagnosis at the start of the study: 26 with BPH and 32 with PCa. In these patients, a portion of urine required to determine ITGAV was collected before the upcoming surgical treatment. Their histological diagnosis was confirmed by postoperative pathomorphological examination. The diagnosis in the remaining 31 patients was determined by examination for an increase in PSA > 4 ng/ml. For these patients needle biopsy of the prostate was performed using a standard 12-cores procedure.

PSA test was available for all participants of the study when clinical stage and Gleason scores were applicable for all PCa cases. Blood test for PSA analysis was performed before the urine collection. To determine the PSA level, the Access-2 analyzer and the HYBRITECH test system (Beckman Coulter Inc.) were used.

The selection of prostate biopsy material was guided by the control of TRUS biopsy, as well as by combining the data of multivariable MRI (mpMRI) with the results of transrectal ultrasound (TRUS) - fusion biopsy. Histological examination of prostate biopsies was performed by a qualified uropathologist. After needle biopsy or receiving post-operative material, a part of prostate tissues was fixed with buffered formalin and processed for routine pathologic diagnosis. The other parts of the prostate tissues were immediately frozen and stored at – 80°C until they were used. The histopathological characteristics of the samples were evaluated after standard preparation of paraffin embedded sections. Diagnosis and Gleason scoring were microscopically confirmed by pathologists.

### Sample Preparation

Human prostate epithelial LnCap, DU145, and PC3 cells were obtained from the American Type Culture Collection. Cells were grown in RPMI 1640 medium (PanEco, Moscow, Russian Federation) supplemented with 10% fetal calf serum and 100 units/ml penicillin and 100 μg/ml streptomycin (PanEco, Moscow, Russian Federation). Cell lysates were prepared by extracting the proteins with a lysis buffer (20 mM Tris, pH 7.5, 100 mM NaCl, 5 mM EDTA, 0.1%Triton X-100), supplemented with protease inhibitor cocktail, set V (Calbiochem). The protein concentrations in each sample were determined by the BCA method (Thermo Fisher Scientific) and equal amounts of protein were used for Western blot.

To prepare conditioned medium (CM), 1×10^6^ LnCaP cells were first plated in complete medium and grown for 24 h. Cells were then washed twice with PBS and placed in a serum-free medium. CM was collected after 48 h, centrifuged at 3,000 rpm to pellet debris, and filtered through a 0.1-micron filter using ultrafiltration toolkit Exo:pure (Prostagnost, Russian Federation) to separate the medium from microvesicles. Then, filtered CM was concentrated with an Amicon ultra 10 centrifugal unit (Millipore). The protein concentration was determined by the BCA method and 20 μg of protein was mixed with a 5× Laemmli gel loading buffer and used for SDS-PAGE gel electrophoresis.

Defrosted urine in the amount of 1.5 ml was centrifuged at 10,000 rpm for 5 min, and 40 μl of the supernatant was then mixed directly with a 5× Laemmli gel loading buffer and used for SDS-PAGE gel electrophoresis.

The needle biopsy tissues were lysed in 200 μl of a lysis buffer described above. The lysates were centrifuged at 12,000 × g for 20 min at 4°C, and the supernatant was collected. Protein concentration was measured with the use of a BCA protein assay kit (Thermo Fisher Scientific). Up to 20 μg of total protein/lane was loaded on 10% SDS-PAGE.

### ITGAV Protein Detection by Western blotting

Proteins were resolved by 10% SDS–polyacrylamide gel electrophoresis and transferred to a nitrocellulose membrane (Bio-Rad). The membranes were blocked for 1 h with 5% (w/v) non-fat milk in a TBST buffer and incubated overnight at 4°C with the rabbit polyclonal anti-ITGAV antibodies (dilution, 1:1,000) or with monoclonal mouse anti-human β-actin (dilution, 1:5,000; Sigma-Aldrich) primary antibodies. Anti-ITGAV rabbit polyclonal antibodies (MBS8508164 MyBioSource or PAB282Hu01 Cloud Clone) were used to identify the ITGAV level. After washing, the membranes were incubated with anti-rabbit peroxidase-coupled secondary antibodies (dilution 1:5,000 in a 5% skim milk–TBST buffer, Rockland). Immunoreactivity was detected with the use of a super signal west pico-chemiluminescent substrate (Thermo Fisher Scientific), and protein bands were visualized with a Fusion Fx imaging station (Vilber Lourmat). The relative level of protein expression was determined by densitometry of the signals using Studio Image Lite software (ver. 5.2).

### Protein Detection in Urine by ELISA

A sandwich enzyme immunoassay (SEB282Hu, Cloud Clone Corp.) with the detection range of 0.156–10 ng/ml was used for quantitative measurements of the ITGAV protein in urine. The standard dilution series, as provided with the kit, was used to generate a standard curve according to which the concentration of ITGAV in each sample was measured. To fit the range of concentrations in the standard curve, a 25-μl aliquot of each urine sample was mixed with 75 μl PBS (dilution 1: 4), and the total volume of 100 μl was used for the assay. All the following procedures were performed according to the manufacturer**’**s protocol. An ELISA kit (SEB673Hu, Cloud Clone Corp.) was used to detect the Insulin growth binding protein 7 (IGFBP7) in 100 μl urine according to the manufacturer**’**s instructions.

### Statistical Analysis

The descriptive statistics and the Student’s t-test of the Excel program were used to obtain the data plots and their p-values. The significance level was evaluated as p < 0.05. The Marley Watkins software (http://edpsychassociates.com/Watkins3.html) was used to generate the ITGAV value curves for receiver operator characteristics (ROC). The area under the ROC curve (AUC) was tested for significance against the null hypothesis that the real area under the ROC curve was 0.5 (i.e. had no diagnostic value). The diagnostic utility statistics from this software package was also used to determine the diagnostic performance values including the sensitivity, specificity, positive predictive power (PPP), and negative predictive power (NPP). The samples were diagnosed as PCa if their serum PSA values were greater than 4 ng/ml (4 ng/ml cutoff). Conversely, they were diagnosed as the benign prostate if those values constituted less than 4 ng/ml. These diagnoses were compared with the pathological diagnoses of subjects. The statistical software was used to find the plots, statistical significance, sensitivity, specificity, PPP and NPP, as well as ROC for the serum PSA, as noted.

## Results

### ITGAV Protein Is Present in Urine Samples of the Non-Cancer Group and Is Not Detected in PCa

**Figure 1:**
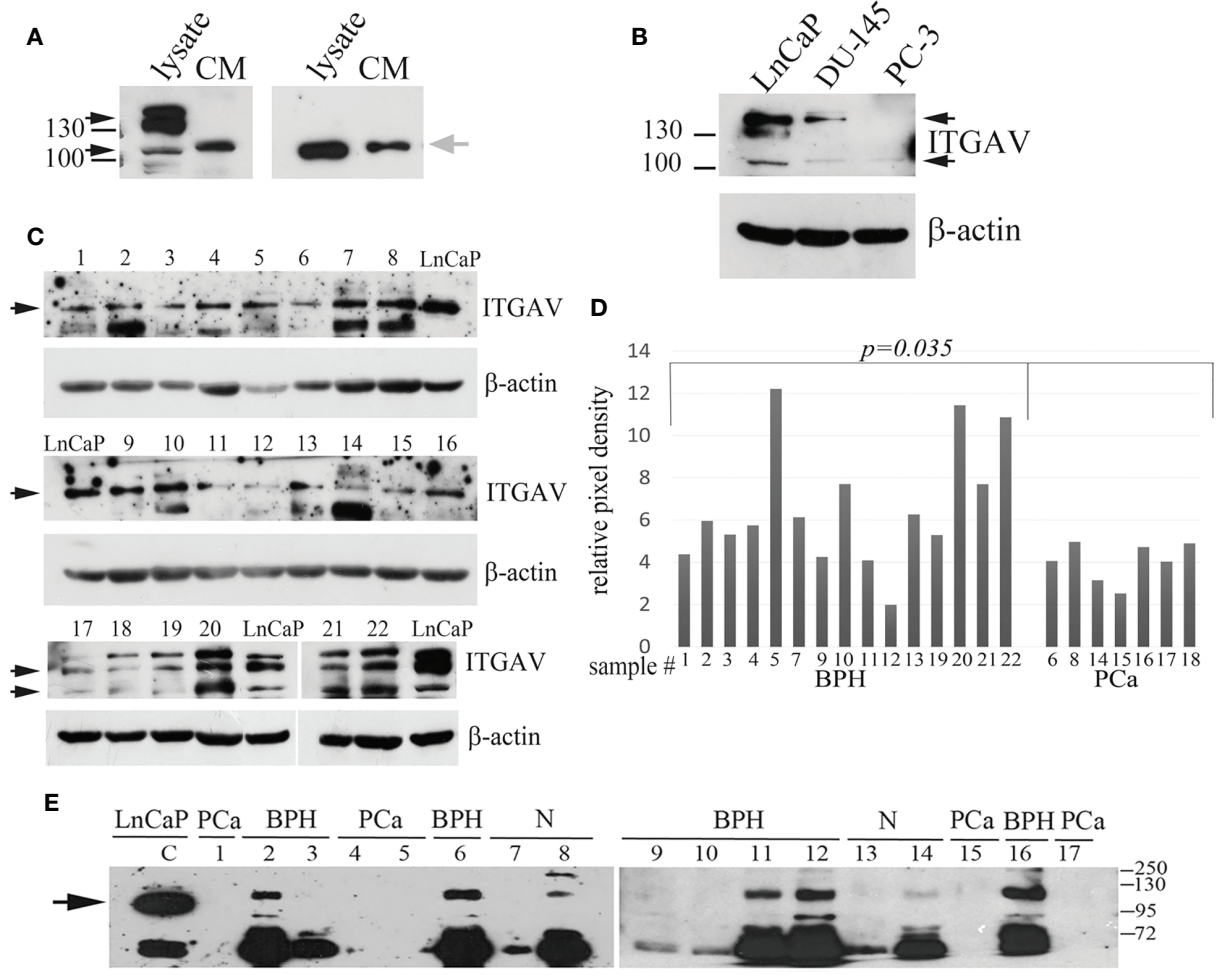
Quantitation of Integrin alpha V (ITGAV) level by Western blot. **(A)** LnCap cell lysates and the conditioned media (CM) produced by LnCaP cells were assayed for ITGAV expression using polyclonal antibodies purchased from Cloud Clone Corp. (A, left panel) and MyBioSource (A, right panel). MW markers of 100 and 130 kDa are shown. Black arrows show ITGAV immunoreactive bands identified in the cell lysates, and grey arrow points on ITGAV found in the CM media. **(B)** Analysis of LnCaP, DU145, and PC3 cell lysates for ITGAV expression using indicated antibodies. β-actin serve as loading controls. Black arrows show the ITGAV-positive bands (about 116 kDa and double band 130–140 kDa), the position of the molecular weight (MW) markers (kDa) are shown on the left. **(C)** ITGAV expression in prostate tissue samples. Samples 1–16 were assayed with anti-ITGAV antibody from MyBioSource, and samples 17–22 were probed with anti-ITGAV antibody from Cloud Clone Corp. Arrows indicate the position of ITGAV immunoreactive bands. β-actin serve as loading controls. **(D)** Quantitation of ITGAV level obtained in C by densitometry analysis. The intensity of the ITGAV signal was normalized to the β-actin level in each sample. *P-value* was calculated by *t-tests* and represent the probability of no difference between ITGAV levels in BPH and PCa samples. **(E)** Urine was collected from patients with prostate cancer (PCa: lines 1, 4, 5, 15, 17) and controls (BPH: lines 2, 3, 6, 9–12, 16; age matched subjects (N): lines 7, 8, and 13, 14). The presence of prostate cancer (PCa) was confirmed by biopsy. Urine samples 1–8 were collected at the N. Lopatkin Scientific Research Institute of Urology; samples 9–17 were from the M.F. Vladimirsky Moscow Regional Research and Clinical Institute and analyzed using anti-ITGAV antibody (MyBioSource). Total proteins in the amount of 30 μg of prostate cell lysate (LnCaP) were used as a control for detection of ITGAV. The arrow points a band corresponding to the predicted MW of ITGAV in LnCaP cell lysate. The position of the MW markers (kDa) is shown on the right.

The predicted molecular weight (MW) for the ITGAV protein is 116 kDa. However, Western blot analysis with anti-ITGAV polyclonal antibodies produced by various companies detect bands corresponding to 116 kDa (My BioSource, Affinity Biosciences, US Biological life sciences) or protein migrating in the position of 130–140 kDa (Cell Signaling Technology, Abcam, BosterBio). The ITGAV gene knockout validated antibodies (Cloud Clone Corp.) and antibodies produced by MyBioSource were used for Western blot analysis to examine the level of ITGAV in cell lysate and the possible presence of the ITGAV in the conditioned media (CM) of cancer cells. Both antibodies revealed the ITGAV in the cell lysate corresponding to the predicted MW (approximately 116 kDa), and antibodies from Cloud Clone Corp. detected an additional double band of 130–140 kDa. However, in the CM of LnCaP cells, both antibodies were able to discover the similar immunoreactive bands migrating a little upper than that identified in cell lysate (**Figure 1A**).

The most commonly used cell lines for prostate cancer study are LnCaP, PC3, and DU145 (18). LnCaP cell line is androgen-sensitive human PCa cell line and its tumorigenicity proved rather poor in athymic nude mice. DU145 and PC3 cells are androgen-independent PCa cells. Orthotopic implantation or intravenous injection of PC3 in athymic nude mice has led to the establishment of lymph node metastases. The DU145 cell line has less metastatic potential compared with PC3 cells. Using western blot analysis, we showed that ITGAV is highly expressed in androgen-dependent LnCaP cells, lesser in DU145 cells, and markedly decreased in androgen-independent, metastatic PC3 cells (**Figure 1B**). To investigate ITGAV expression in the PCa and BPH tissues the analysis of the needle biopsy specimens was performed. Although the ITGAV level was varied in BPH samples, a statistically significant decrease was noted in the cancer tissue samples as compared with the BPH tissues (**Figures 1C, D** **)**.

Because “soluble” ITGAV protein was found in the CM media of LnCaP cells, we examined the levels of ITGAV in the urine to address its possible presence in biological fluid. The randomly chosen urine specimens were collected from the healthy individuals and patients with the BPH or PCa diagnoses and analyzed by Western blotting in a blinded fashion with the use of anti-ITGAV antibodies (MyBioSource) and LnCaP cell lysate as a control for immunoreactive band position. A band migrating upper than that of 116 kDa and an additional band (approximately 72 kDa) were detected in the majority of the urine samples of the control group (**Figure 1E**). The lower MW immunoreactive band could be the result of ITGAV proteolysis. However, both bands were not observed in the urine of PCa patients. Notably, that “soluble” ITGAV produced by cultured cells and ITGAV in the urine of non-cancerous specimens seems to have similar MW because both have the same mobility on SDS PAGE but migrating differently than the intracellular ITGAV of 116 kDa.

Finally, our data indicate that the ITGAV protein is secreted into urine, and BPH vs. PCa cases can be distinguished by the validation of the ITGAV level. This is why a further quantitative study of urinary ITGAV expression was performed to estimate its efficacy in PCa diagnosis.

### The use of ITGAV Protein Level in Urine for Prostate Cancer Diagnosis

**Figure 2:**
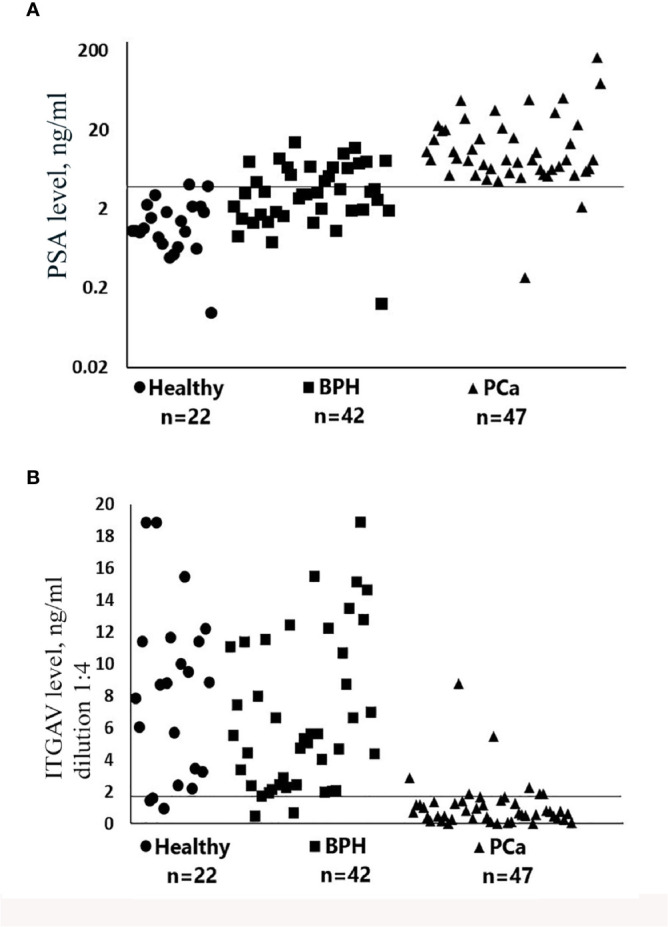
Scatter plots representing serum prostate-specific antigen (PSA) **(A)** and urinary Integrin alpha V (ITGAV) **(B)** concentrations determined by enzyme-linked immunosorbent assay (ELISA). The number of samples (n) for each group of subjects is shown. Patient group abbreviations are described in **Table S1**. The line on each scatter plot indicates a cutoff value of 4 ng/ml for serum prostate-specific antigen (PSA) **(A)** and 2 ng/ml for urinary ITGAV **(B)**. Samples of urine for the detection of ITGAV were diluted 1:4 as described in *Materials and Methods*.

Urine samples obtained from 111 patients at two hospitals were diagnosed to investigate whether ITGAV in patient urine could be used to distinguish between PCa and the benign prostate. The level of urinary ITGAV was quantified by ELISA. The diagnoses of the patients were based on their serum PSA levels, and pathological analyses of biopsy were performed for the PCa and BPH groups. The age of the patients in this study was similar to the former (51 to 79 years). As it was expected, the prostate cancer patients had significantly higher serum PSA than the BPH and age-matched control subjects when the 4 ng/ml cutoff for PSA detection was used (**Figure 2A** **)**. As depicted in **Figure 2B**, the ITGAV level was significantly lower in the PCa urine samples. The mean values of 8.28 and 6.77 ng/ml, respectively, in the age-matched controls and BPH samples decreased to 1.14 ng/ml in the PCa samples (p < 0.00001). Thus, the mean ITGAV concentration was 5.9-fold lower in the PCa patients as compared with that in the BPH and healthy control groups. The summary of the characteristics of the urine sample cohort is represented in **Table S1**. The ITGAV decrease was detected in 43 of 47 (91.5%) individuals with PCa when 2 ng/ml cutoff was used.

Notably, in two individuals in this PCa group, the serum PSA was low and constituted less than 2.2 ng/ml (**Table S2**). In the BPH individuals and in the age-matched control group, ITGAV was decreased in 5 of 42 (11.9%) and in 3 of 22 (13.6%), respectively. It should be noted that in the BPH group (confirmed by biopsy) 16 of 42 (38%) individuals had the increased serum PSA (4.6 to 14.6 ng/ml). The “normal” urinary ITGAV level (4.0–11.4 ng/ml) was detected in nine individuals from this group, and the ITGAV concentration close to the cutoff value of 2 ng/ml (2.04–2.91 ng/ml) was found in the other seven. These data indicate that the urinary ITGAV test can clearly predict the benign diagnosis in 56% cases with the abnormal level of the serum PSA. We found no significant correlation between the urinary ITGAV and the serum PSA levels, as well as that between the ITGAV concentration and the combined Gleason score of 6, 7, or 8 (**Table S2**).

**Figure 3:**
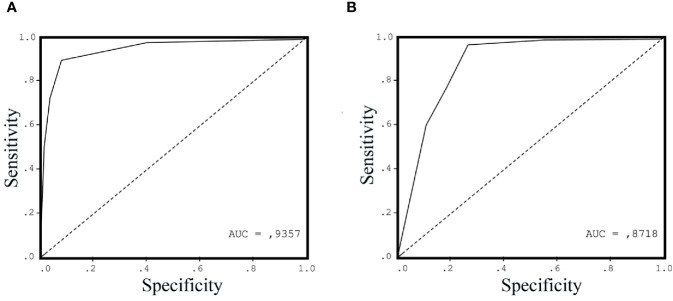
Receiver operating characteristic curve (ROC) for urinary Integrin alpha V (ITGAV) **(A)** and serum prostate-specific antigen (PSA) **(B)** levels in patients with prostate cancer (PCa) vs. control group subjects. The area under the curve (AUC) is shown for each ROC analysis, making 0.9357 for urinary ITGAV and 0.8718 for serum PSA.

The result of ROC analysis showed that AUC of the ROC curve for urinary ITGAV at the cutoff value of 2 ng/ml was 0.93 (**Figure 3A**), while AUC for the serum PSA at the cutoff value of 4 ng/ml was 0.87 (**Figure 3B**). The urinary ITGAV parameters to distinguish PCa from the benign prostate were sufficiently high with 91.5% sensitivity, 91.4% specificity, 87.7% PPP, and 94.1% NPP. The serum PSA also had the high sensitivity of 95.8%, however its specificity and PPP were lower, respectively, 71.4% and 71.8%, as compared with ITGAV. The NPP value (95.7%) of the serum PSA was compatible with that of ITGAV. These data demonstrate the potential of urinary ITGAV as a relevant test for prostate cancer diagnosis.

We also analyzed abundance of another urinary protein, the insulin growth factor binding protein (IGFBP7), to distinguish between two possible scenarios: association of PCa pathology with reduction in the pool of secreted proteins and, otherwise, specificity of the urinary ITGAV low level to the disease. IGFBP7 is the known urinary prognostic marker in early acute kidney injury (19, 20). Urine samples were randomly chosen from the cohort who was previously used for the ITGAV identification (**Figure S1**). There was no statistically significant difference between the benign control group and the PCa group in the IGFBP7expression (p = 0.66).

### ITGAV Variability in Urine Samples. Importance of the Procedure of Sample Collection for the Determination of the ITGAV Level

The clinical urine samples used in this study were donated without a prostate massage, because we suggested that proteins secreted by prostate cells could be present in the first pass of urine. To compare the ITGAV level and investigate how important is the sample collection procedure 10–20 ml of the first and second urine passes were collected from the same individual followed by ITGAV and IGFBP7 measurements by ELISA. As shown in **Figures S2A, B**, in all 21 samples, the ITGAV level was significantly lower in the second pass as compared with the first urine pass (p = 0.003), while the IGFBP7 protein level was similar in the both passes. In 12 of 21 (57%) second pass samples and only in two of 21 (9.5%) first pass samples, urinary ITGAV was found below the cutoff value of 2 ng/ml (**Figure S2A**). The observation that the ITGAV level is lower in the second urine pass suggests false PCa prediction in case if the second urine pass instead of the first pass is donated. The low ITGAV level in the first urine pass from the healthy subjects (9.5%) was compatible with the values observed in the clinical samples of the BPH and age-matched control groups (11.9% and 13.6%, respectively; **Figure 2B** **)** reflecting variability in protein expression among individuals. The ITGAV enrichment in the first urine pass suggests its presence in prostate fluid. However, an additional analysis using urine samples before and after prostatectomy is necessary to clarify can this protein be considered as a prostate-specific marker?

## Discussion

In this study, we showed that ITGAV, the member of the integrin receptor family, is present as a “soluble” protein in the CM produced by prostate cancer cells and can be found in first pass urine collected without preceding prostate massage. These results represent the “proof of concept” of the use of ITGAV level for liquid biopsy for PCa detection. The majority of PCa patients have the lower ITGAV level in urine as compared with the non-cancer controls. Our data indicate that ITGAV found in urine with the high 91.5% sensitivity and 91.4% specificity, when the cutoff value equals 2 ng/ml, can be used to discriminate between PCa and the benign prostate.

Currently, PSA is the useful tool for the early detection and monitoring of PCa nevertheless, PSA values are frequently vary depending on prostate volume and age. Other researchers showed on a large cohort of subjects with serum PSA > 4 ng/ml that only 25% individuals, who had prostate biopsy, suffered from PCa (21). Thus, the low sensitivity of the PSA test leads to false positive results and many unnecessary biopsies. Moreover, PCa is often present in men with serum PSA below the cutoff level greater than 4 ng/ml (22). Our study revealed that the urinary ITGAV test can clearly distinguish between PCa and BPH and predict the benign diagnosis in 56% cases with the serum PSA level greater than 4 ng/ml, suggesting its use to reduce unnecessary biopsies in case of PCa diagnosis.

Urine represents an ideal source of biomarkers due to its anatomic proximity to the prostate gland because it can be obtained noninvasively. Recently available urine biomarkers based on differential gene expression are sufficiently complex and require, in large, urine collection after prostate massage, because these tests, such as PCA3, are based on analysis of RNA molecules extracted from cells expelled into urine (23). Conversely, ITGAV can be revealed in unprocessed urine when the cellular component of collected urine is removed. In this case, only 25 μl supernatant is required to quantitate an ITGAV level with the use of the simple ELISA test.

We found that LnCaP androgen-sensitive prostate cancer cells express a higher level of ITGAV than androgen-insensitive and more aggressive cancer cells DU145 and PC3. This observation suggests that the lack of androgen signaling can lead to dysregulation of the ITGAV expression. This remains to be directly evaluated including the analysis of ITGAV in urine samples and biopsies collected from patients with PCa resistant to androgen suppression therapy.

The transmembrane ITGAV receptor consists of 1048 amino acids (aa), from which aa 31-992 compose the extracellular domain. ITGAV has 13 predicted sites for glycosylation and this post-translational modification can decrease its mobility on SDS-PAGE gels leading to the detection of the heavy band(s) with MW 130-140 kDa. The appearance of “soluble” ITGAV in the CM of cultured cells and urine can be the result of proteolytic cleavage of the extracellular domain by proteases. Recently published data have shown that transmembrane glycoprotein e-cadherin is cleaved by proteases to generate a soluble ectodomain fragment, termed sEcad which can be released in the cell culture medium or the sera in response to different stimulus (24, 25). Currently, the mass spectrometry-based identification of the amino acid sequence of the “soluble” ITGAV is in our ongoing study.

Recently published studies dedicated to other potential urinary protein biomarkers have shown that levels of some of them (EN2, αHGF, IGFBP3, ZAG) are elevated in PCa, while the urinary ANXA3, PAP, and PSA have inverse relationship with cancer (8, 11, 12, 26–29). Moreover, unlike the serum PSA whose level is elevated in PCa, the urinary PSA level is decreased. This allowed suggesting the latter as a prognostic marker in discrimination between prostate cancer and BPH, particularly, when serum PSA abundance is 2.5–10 ng/ml (29).

Immunohistochemical studies revealed deregulation of integrin expression in the case of PCa progress to advanced stages, in the similar way, the expression of α subunits was shown to be downregulated in prostate cancer tissues (30). The TMA studies on 1284 prostate cancer patients have indicated that cytoplasmic ITGAV immmunostaining is usually weak with no significant differences among different Gleason scores. At the same time, membranous immunostaining of αv-pan is decreased with PCa progression, which is significantly inversely correlated with Gleason scores (15). We, as far, do not know whether the decreased level of ITGAV in the urine samples of the PCa patients is related to downregulation of integrin expression in membranes of cancer cells, or it is related to neoplastic rearrangements of prostate gland leading to disruption of prostatic ducts. This can result in altered ITGAV secretion into prostate fluid and, finally, in altered first void urine. Our preliminary analysis of the cancer biopsy specimens (**Figures 1C, D**) assumes that the ITGAV lower level in urine can be a consequence of downregulation of its expression in PCa tissues. However, more biopsy samples are required to expand this study. Moreover, studies of individual urinary ITGAV levels in the case of other urothelial cancers (bladder, renal, and ureteric) should be included in this overall project to define this biomarker specificity for the prostate cancer.

In conclusion, ITGAV protein is found in the CM produced by cancer cells and the urine. ITGAV expression is reduced in aggressive androgen-insensitive cancer cells and in the urine of the PCa patients. Urinary ITGAV can be suggested as the complement to the serum PSA used in the diagnosis of individuals with low tract urinary symptoms, or a general screening of the entire population. This follows from the diagnosis simplicity, which does not require additional procedures such as prostate massage. The ELISA-based technique used in our study potentially allows designing several different assays based on ITGAV (alone or in combination with other urinary protein markers).

## Data Availability Statement

The original contributions presented in the study are included in the article/**Supplementary Material**. Further inquiries can be directed to the corresponding author.

## Ethics Statement

The studies involving human participants were reviewed and approved by Ethics Committees of the N.A. Lopatkin Research Institute of Urology and Interventional Radiology and the M.F. Vladimirsky Moscow Regional Research and Clinical Institute (MONIKI), Moscow, Russian Federation. Before the study, participants signed an informed consent form.

## Author Contributions

MZ: Conceptualization and supervision, investigation, data curation, formal analysis and validation, and writing—original draft preparation. MM: Investigation and data curation. AS: Funding acquisition and resources. JP: Investigation and data curation. AS: Investigation and data curation. KS: Formal analysis and validation, funding acquisition and resources, and writing—original draft preparation. All authors contributed to the article and approved the submitted version.

## Funding

This study was supported by the Foundation for Assistance to Small Innovative Enterprises (FASIE), Russian Federation (project no. 2432GS2/22671) granted to MZ.

## Conflict of Interest

Author MZ and KS were employed by company Prostagnost.

The remaining authors declare that the research was conducted in the absence of any commercial or financial relationships that could be construed as a potential conflict of interest.
