# Supplementary material for: Integrin Alpha V in Urine: A Novel Noninvasive Marker for Prostate Cancer Detection
Source: Front Oncol. 2021 Mar 10;10:610647. doi: 10.3389/fonc.2020.610647 (PMC8006463; doi:10.3389/fonc.2020.610647)
Supplement: Supplementary file 1 [file DataSheet_1.docx]

Supplementary Material

# Supplementary Data

**Supplementary Table 1.** Results from sample analysis of all patients. Values of p showed a statistical difference between the serum PSA and urinary ITGAV levels of prostate cancer patients, benign prostatic hyperplasia patients, and age-matched controls.

| Patient group | Age, years; average and range | Serum PSA level, ng/ml; median and range; p = 0.001 | Urinary ITGAV, ng/ml; median and range; p = 2.42·10–11 |
| --- | --- | --- | --- |
| Prostate cancer n = 47 | 64 (51–76) | 18.7 (0.28–171) | 1.14 (0.06–8.8) |
| Benign prostatic hyperplasia (BPH) n = 42 | 64 (53–79) | 4.4 (0.13–14.6) | 6.77 (0.5–18.9) |
| Age-matched control subjects n = 22 | 58 (51–68) | 1.575 (0.1–4.2) | 8.28 (1.0–18.9) |

**Supplementary Table 2.** Patient demographics and urine screening results using an ELISA to quantify the levels of urinary ITGAV and serum PSA.

| Ng/No. | Age | PSA ng/ml | ITGAV ng/ml | Gleason score |
| --- | --- | --- | --- | --- |
| **Healthy** | | | | |
| 1 | 58 | 1,1 | 7,93 |  |
| 2 | 58 | 1,1 | 6,10 |  |
| 3 | 51 | 1,04 | 11,48 |  |
| 4 | 66 | 1,19 | 18,94 |  |
| 5 | 56 | 2,33 | 1,51 |  |
| 6 | 56 | 1,6 | 1,68 |  |
| 7 | 61 | 3,12 | 18,94 |  |
| 8 | 59 | 0,92 | 8,78 |  |
| 9 | 56 | 0,75 | 1,01 |  |
| 10 | 66 | 1,88 | 8,85 |  |
| 11 | 53 | 0,5 | 11,75 |  |
| 12 | 66 | 0,55 | 5,77 |  |
| 13 | 51 | 0,69 | 2,49 |  |
| 14 | 58 | 1,45 | 10,08 |  |
| 15 | 53 | 1,08 | 15,53 |  |
| 16 | 59 | 4,2 | 9,57 |  |
| 17 | 68 | 2,24 | 2,28 |  |
| 18 | 55 | 0,66 | 3,50 |  |
| 19 | 56 | 2,25 | 11,48 |  |
| 20 | 56 | 1,9 | 3,31 |  |
| 21 | 61 | 4 | 12,30 |  |
| 22 | 53 | 0,1 | 8,95 |  |
| **BHP** | | | | |
| 23 | 53 | 2,24 | 11,13 |  |
| 24 | 54 | 0,94 | 5,56 |  |
| 25 | 63 | 1,57 | 7,46 |  |
| 26 | 68 | 3,29 | 3,43 |  |
| 27 | 75 | 8,2 | 11,42 |  |
| 28 | 78 | 1,4 | 4,48 |  |
| 29 | 64 | 4,56 | 2,39 |  |
| 30 | 57 | 1,76 | 0,51 |  |
| 31 | 68 | 3,39 | 8,03 |  |
| 32 | 68 | 1,42 | 1,78 |  |
| 33 | 62 | 0,79 | 11,58 |  |
| 34 | 60 | 1,9 | 1,97 |  |
| 35 | 62 | 9,02 | 2,18 |  |
| 36 | 55 | 1,68 | 6,68 |  |
| 37 | 60 | 6,93 | 2,45 |  |
| 38 | 65 | 5,67 | 2,91 |  |
| 39 | 60 | 14,6 | 2,29 |  |
| 40 | 66 | 2,8 | 12,49 |  |
| 41 | 62 | 3,19 | 0,69 |  |
| 42 | 70 | 3,2 | 2,48 |  |
| 43 | 67 | 7,2 | 4,76 |  |
| 44 | 69 | 1,4 | 5,37 |  |
| 45 | 65 | 3,32 | 5,14 |  |
| 46 | 67 | 2,09 | 5,66 |  |
| 47 | 57 | 4,6 | 15,53 |  |
| 48 | 64 | 5,41 | 5,67 |  |
| 49 | 62 | 7,02 | 4,08 |  |
| 50 | 69 | 1,1 | 2,00 |  |
| 51 | 59 | 3,67 | 12,30 |  |
| 52 | 68 | 10,42 | 2,04 |  |
| 53 | 74 | 6,8 | 2,13 |  |
| 54 | 54 | 2 | 4,71 |  |
| 55 | 67 | 12,3 | 10,71 |  |
| 56 | 79 | 7,75 | 8,80 |  |
| 57 | 50 | 2,03 | 13,53 |  |
| 58 | 66 | 8,3 | 6,69 |  |
| 59 | 67 | 3,46 | 15,20 |  |
| 60 | 59 | 3,67 | 18,94 |  |
| 61 | 65 | 2,7 | 12,83 |  |
| 62 | 57 | 0,13 | 14,71 |  |
| 63 | 66 | 8,31 | 7,05 |  |
| 64 | 59 | 2 | 4,42 |  |
| **Prostate cancer** | | | | |
| 65 | 68 | 11 | 2,93 | сT2cN0M0G6(G3+3) |
| 66 | 65 | 8,6 | 0,75 | сT2аN0M0 (G3+4) |
| 67 | 66 | 15,6 | 1,22 | сT2bN0M0 (G3+4) |
| 68 | 76 | 23 | 1,22 | сT3bN0M0(G4+4) |
| 69 | 65 | 20 | 1,05 | сT2aN0M0 (G3+4) |
| 70 | 64 | 20,65 | 0,45 | T3bN0M0 (G3+4) |
| 71 | 65 | 5,43 | 0,22 | T2aN0M0 (G4+3) |
| 72 | 58 | 10,89 | 1,41 | T2cN0M0 (G3+3) |
| 73 | 53 | 9 | 0,52 | Т2N0M0 (G3+3) |
| 74 | 66 | 48,4 | 0,23 | T2cN0M0 (G4+3) |
| 75 | 69 | 28,6 | 0,53 | cT2cN0M0 (G3+4) |
| 76 | 53 | 8,5 | 0,08 | Т3bN0M0 (G4+3) |
| 77 | 61 | 11,64 | 0,30 | сТ3bN0M0 (G3+3) |
| 78 | 58 | 5,48 | 1,31 | T3aN0M0 (G4+3) |
| 79 | 65 | 16 | 8,80 | T2cN0M0 (G3+3) |
| 80 | 65 | 7,52 | 1,48 | T2cN0M0 (G4+3) |
| 81 | 59 | 4,91 | 0,86 | T2aN0M0 (G3+3) |
| 82 | 56 | 6,66 | 1,91 | T2cN0M0 (G3+4) |
| 83 | 55 | 36,3 | 0,34 | T3bN0M0 (G4+4) |
| 84 | 59 | 4,7 | 1,03 | T1cN0M0 (G3+3) |
| 85 | 66 | 21,4 | 1,73 | T2cN0M0 (G4+4) |
| 86 | 65 | 8,6 | 1,21 | T3аN0M0 (G 3+3) |
| 87 | 60 | 5,87 | 0,47 | T2cN0M0 (G3+3) |
| 88 | 69 | 16,2 | 0,18 | T2cN0M0 (G4+3) |
| 89 | 67 | 8 | 5,50 | T2аN0M0 (G4+4) |
| 90 | 64 | 5,14 | 0,08 | сT2аN0M0 (G3+3) |
| 91 | 73 | 0,28 | 1,51 | сT1N0M0 (G4+4) |
| 92 | 58 | 49,3 | 1,71 | T3аN0M0 (G4+4) |
| 93 | 57 | 8,55 | 0,08 | сT1сN0M0 (G3+3) |
| 94 | 67 | 10,59 | 0,14 | T2cN0M0 (G3+4) |
| 95 | 68 | 6,43 | 1,29 | T2cN0M0 (G4+3) |
| 96 | 67 | 5,63 | 0,71 | сT2cN0M0 (G3+3) |
| 97 | 55 | 5,4 | 0,60 | T3bN0M0 (G5+5) |
| 98 | 68 | 6,52 | 0,58 | T2bN0M0 (G3+3) |
| 99 | 70 | 34,16 | 2,29 | сT2cN0M0 (G3+3) |
| 100 | 57 | 7,2 | 0,06 | T2сN0M0 (G3+4) |
| 101 | 70 | 51,58 | 0,59 | сT2сN0M0 (G3+4) |
| 102 | 66 | 8,7 | 1,89 | сT2bN0M0 (G3+3) |
| 103 | 62 | 13,68 | 1,93 | сT2bN0M0 (G3+4) |
| 104 | 70 | 5,54 | 0,88 | рT3аN0M0 (G4+3) |
| 105 | 51 | 24 | 0,82 | сT3аN0M0 (G3+5) |
| 106 | 55 | 2,17 | 0,51 | сT2сN0M0 (G3+3) |
| 107 | 59 | 6,13 | 0,36 | сT2сN0M0 (G3+3) |
| 108 | 68 | 6,67 | 0,83 | сT2bN0M0 (G3+3) |
| 109 | 76 | 8,7 | 0,30 | T2сN0M0 (G3+3) |
| 110 | 71 | 171 | 0,65 | T2cN0M0 (G4+3) |
| 111 | 72 | 80 | 0,12 | T2cN0M0 (G4+3) |

*
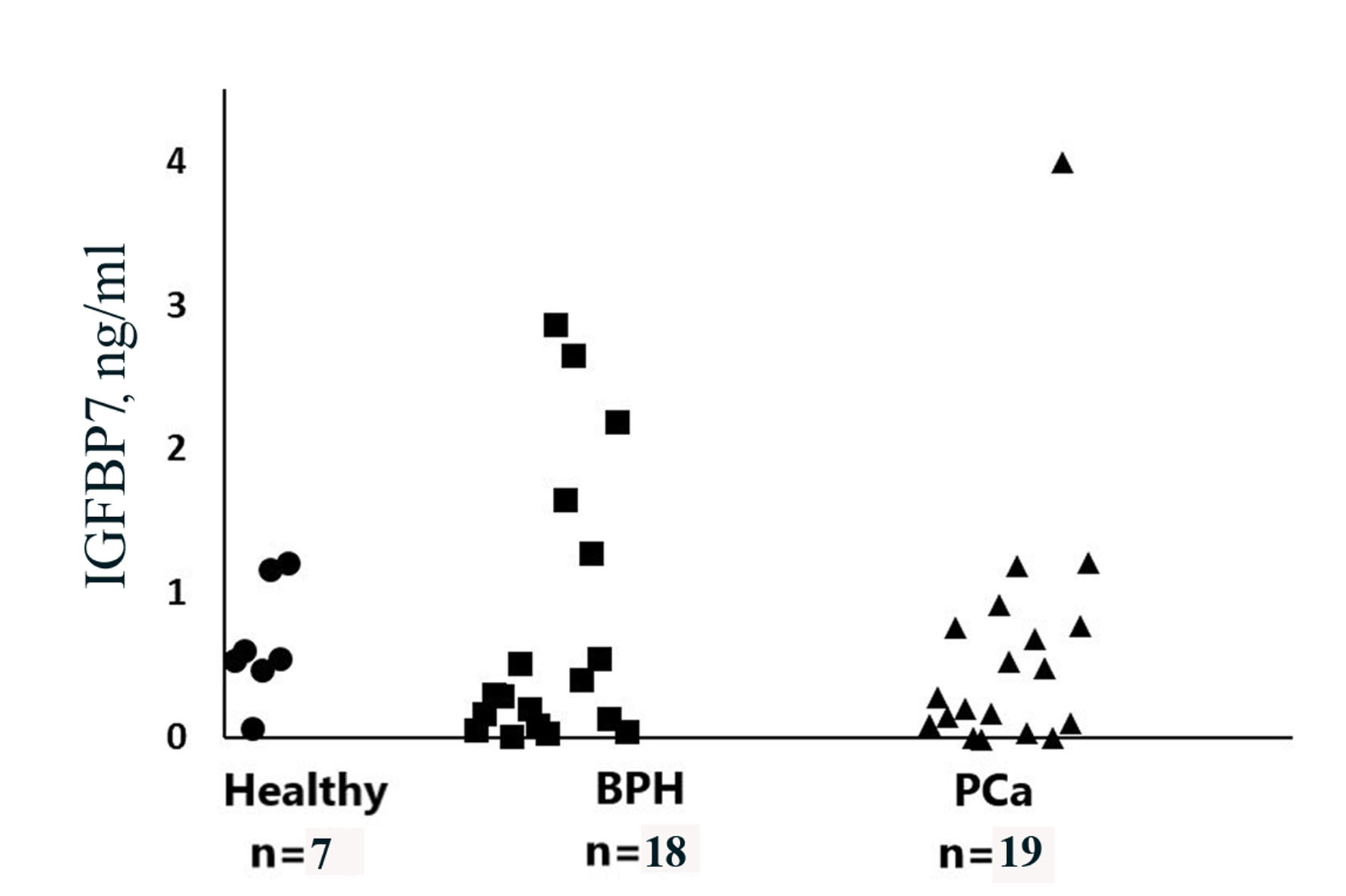
*

**Supplementary Figure 1.** A scatter plot representing the IGFBP7 ELISA results for randomly chosen urine samples from a cohort, which previously was used for ITGAV identification. There is no statistically significant difference between the benign control group and PCa in the level of IGFBP7 (p > 0.05).

*
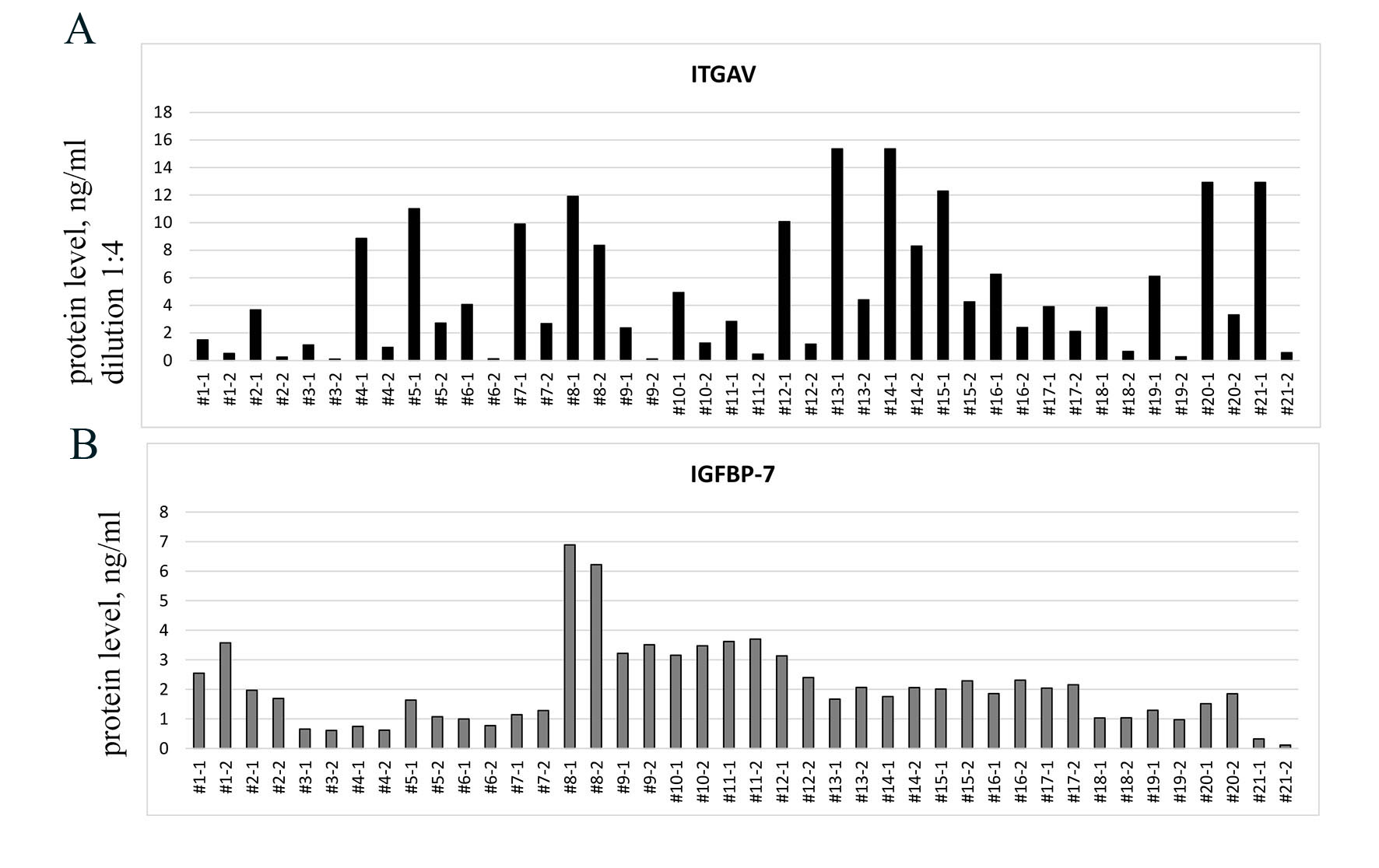
*

**Supplementary Figure 2.** A comparison of ITGAV (A) and IGFBP7 (B) concentrations between the first (#n-1) and (#n-2) passes of urine from healthy individuals. The difference in values between the first and second pass of urine for ITGAV is statistically significant (p < 0.05) while there is no difference for IGFBP7 protein level (p > 0.05) in both passes.
